# The Austrian Public Health Week & the European Public Health Week: Thomas Dorner

**DOI:** 10.1093/eurpub/ckac129.288

**Published:** 2022-10-25

**Authors:** T Dorner

**Affiliations:** Public Health Association Austria, Vienna, Austria

## Abstract

How did the Austrian Public Health Week start and what does it have to do with the European Public Health Week?

